# Zambia Assessment of Tuberculosis (TB) and HIV in the Mines (ZATHIM): implications for programs and policies

**DOI:** 10.1186/s12889-022-13053-8

**Published:** 2022-04-20

**Authors:** Laura Jean Podewils, Elizabeth F. Long, Tyler J. Fuller, David Mwakazanga, Kelvin Kapungu, Mathias Tembo, Sydney Mwanza, Kathryn G. Curran, Jonathan P. Smith, James L. Tobias, Webster Kasongo

**Affiliations:** 1 Centers for Disease Control and Prevention, Atlanta, GA USA; 2 Emory University Rollins School of Public Health, Atlanta, GA USA; 3 Tropical Diseases Research Centre, Ndola, Zambia; 4 School of Public Health, Yale University, New Haven, CT USA; 5 Peraton, Atlanta, GA USA

**Keywords:** Miners, Mining, Healthcare, Africa, KAPs, Policy, TB, HIV

## Abstract

**Background:**

Mineworkers in Southern Africa have the highest rates of tuberculosis (TB) among working populations in the world (The World Bank, Benefits and costs associated with reducing tuberculosis among Southern Africa’s mineworkers, 2014), making mineworkers a key population for TB program efforts. The current evaluation aimed to characterize mineworkers and former (ex-) mineworkers, and assess knowledge, attitudes and practices related to TB and HIV care among mineworkers and healthcare workers (HCWs) in Zambia.

**Methods:**

A mixed-methods evaluation of current and former (ex-) mineworkers and HCWs was conducted in the Copperbelt and North-Western provinces, Zambia. Knowledge, attitudes and practices (KAPs) related to TB care and policies were assessed using a structured survey. Focus Group Discussions (FGDs) were conducted with current and ex-mineworkers to understand perceptions, practices, and barriers related to accessing healthcare for TB.

**Results:**

Overall, 2,792 mineworkers and 94 HCWs completed the KAP survey, and 206 (171 current, 71 ex-) mineworkers participated in FGDs. Mineworkers and ex-mineworkers were knowledgeable about TB symptoms (cough; 94%), transmission (81.7%) and treatment (99.2%). Yet, barriers to seeking care were evident with 30% of mineworkers experiencing cough, and 19% reporting 2 or more TB symptoms at the time of the survey. The majority of mineworkers (70.9%) were aware of policies barring persons from working after a diagnosis of TB, and themes from FGDs and HCW comments (*n* = 32/62; 51.6%) recognized fear of job loss as a critical barrier to providing timely screening and appropriate care for TB among mineworkers. The majority (76.9%) of mineworkers indicated they would not disclose their TB status to their supervisor, but would be willing to share their diagnosis with their spouse (73.8%).

**Conclusion:**

Fear of job loss, driven by governmental policy and mistrust in mining companies, is a major barrier to healthcare access for TB among mineworkers in Zambia. As a result of these findings, the government policy prohibiting persons from working in the mines following TB disease is being repealed. However, major reforms are urgently needed to mitigate TB among mineworkers, including ensuring the rights of mineworkers and their communities to healthy living and working environments, improved social responsibility of mining companies, and facilitating choice and access to affordable, timely, and high-quality healthcare services.

Tuberculosis (TB) is a leading cause of infectious death globally [1]. Examination of TB in the Southern African region has revealed that conditions associated with minework directly amplify TB transmission and lend to the highest incidence rates among working populations in the world [2, 3]. Zambia has one of the highest burdens of TB in the world, estimated at 346/100,000 in 2019 [1]. Over half (60%) of persons with TB disease in Zambia also have HIV. The mining industry is an essential part of the economy in Zambia [4], with over 60,000 individuals working the Zambia mines in the Copperbelt and North-Western provinces [5]. The Copperbelt province, which contains most of the mines in Zambia and the vast majority of Zambian mineworkers, has both the highest burden of TB [6] and HIV in the country [7]. TB is a leading cause of death among mineworkers and former mineworkers, causing over 20% of deaths overall and over 40% of deaths among persons living with HIV [8]. In fact, TB related deaths exceed the proportion of deaths due to mining accidents in this population [9].

Multiple factors contribute to an increased risk for TB among mineworkers: exposure to silica dust and gases that may compromise lung function; working and living in crowded conditions; increased rates of HIV; and the transient and migratory patterns of mineworkers driven by a dire economic need to support individuals and their families from communities across the country [10–14]. Zambia has experienced a burgeoning expansion of mines and the mining industry in recent years into the North-Western province [5]. Mineworkers in Zambia are employed by one of two mechanisms: directly by the mining company or through a contracting agency; the proportion that are contracted is expanding with increased foreign investment in the mining sector. Healthcare benefits differ according to the method of employment. Mineworkers in Zambia who are diagnosed with TB are prohibited from ever working in the mines again as a result of a 1999 policy (The 1999 Workers’ Compensation Act), originally developed as a form of infection control [15, 16]. Restrictions like this may create a strong disincentive for mineworkers to seek TB diagnosis and treatment. Together, these factors may impact the composition of the mining workforce and healthcare for TB in mining communities. There is limited research on the impact of the TB and TB/HIV epidemic on the mining community.

We aimed to characterize mineworkers in Zambia and better understand both mineworkers’ and healthcare workers’ knowledge, attitudes, and practices related to TB and HIV disease. Additionally, we sought to understand healthcare for TB disease in Zambia. Identifying best practices, key challenges, and preferences for care among mineworkers will help guide the design and implementation of programmatic interventions aimed at reducing the burden of TB and HIV in the mining community.

## Methods

### Study design

We conducted a mixed-methods cross-sectional evaluation of current and former (ex-) mineworkers and healthcare workers at government and mining-owned health facilities in the Copperbelt and North-Western provinces, Zambia.

### Data collection

Trained study staff interviewed current and ex-mineworkers using a structured survey to obtain knowledge, attitudes, and practices (KAPs) related to TB; these were augmented by focus group discussions (FGDs) seeking to gain a thorough understanding of perspectives and practices related to health-seeking behavior, access to care, and barriers to care. Staff interviewed healthcare workers using semi-structured surveys pertaining to the provision of TB care for mineworkers. Data were collected from seven Districts in the Copperbelt Province: Chambishi, Chilalabombwe, Kalulushi, Kitwe, Luanshya, Lufwanyama, and Mufulira and three towns (Kalumbila, Lumwana, and Solwezi) in the Solwezi District in the North-Western Province. All data collection activities were conducted between October 2017 and January 2018.

### Sampling and recruitment

#### Mineworkers and ex-mineworkers

Population propotional to size sampling was used to derive a target population of mineworkers and ex-mineworkers for the KAP surveys. Total population estimates for each of the towns with mining activities in the 2 predominant mining provinces, Copperbelt and North-Western, were obtained from the Zambia Census [17]. Half (50%) of this total population, based on consultation with government, mining companies and industry experts, was estimated as the population with current or former employment with the mines. We aimed to sample 2% of the total mineworking population and applied the proportion contribution from each mining town for a total of 2,401 total mineworkers. Assuming a 10% refusal rate, we calculated a sample of 2,641 mineworkers (2,401 X 110%) and rounded it to a total target sample size of 2,700; estimates of 60% and 40% as the proportion of current and ex-mineworkers were applied based on data from mining administrative offices, respectively [(1,620 current (60%), 1,080 ex (40%).] Of the 2,700 current and ex-mineworkers targeted for inclusion in the KAP survey, 1,700 were from the Copperbelt province and the 1,000 from North-Western province.

In addition, we aimed to conduct three FGDs (one each with current mineworkers, ex-mineworkers and either female or younger [aged 18–30 years] mineworkers), each comprised of 8–12 participants, per mining district. FGDs with female or younger participants were carried out specifically and separately to ensure that the voices of these members of the mining community, though comprising a small proportion of overall labor, were included without the influence of the majority of older, male mineworkers.

Convenience sampling was used for the mineworker KAP surveys and FGDs. Study staff partnered with mining companies, health facilities, community/civic leaders, and community health workers to promote awareness of the study and identify potential participants. Staff communicated information about the study and details about central meeting points for community members who were interested in participating. Mineworkers and ex-mineworkers were eligible for participation if they were currently working or previously worked in the mines for at least a one-year period. Each person was recruited to participate in either the FGD or the KAP survey dependent on the space available in upcoming FGDs. If the FGD was full, already completed in the area, or the participant was not interested, they were invited to complete the KAP survey. Research assistants assessed potential participants for eligibility and obtained written informed consent from persons meeting eligibility criteria.

#### Healthcare workers

A list of all government and mining-operated health facilities located in the mining communities was obtained from the Zambia Ministry of Health. The TB coordinator from each facility in mining districts (*n* = 94) of the Copperbelt and North-Western provinces was invited to participate in the semi-structured interview.

#### Analysis

The study populations of the mineworkers and healthcare workers completing the KAP survey, interviews, and FGDs were summarized using descriptive statistics. Frequencies were calculated for categorical variables and medians and interquartile ranges (IQR) were calculated for continuous variables. Differences between distributions by current mineworker vs. ex-mineworkers, Copperbelt vs. North-Western province, and mining-employee vs. contract employee were assessed using *p*-values based on chi-square tests for categorical variables and the Wilcoxon rank-sum test for differences in medians of continuous variables. An alpha level of 0.05 was used to determine statistical significance. Quantitative analyses were conducted using Stata 15.0 (College Station, Texas).

The FGDs were reviewed and analyzed using a thematic qualitative approach based on the Health Belief Model [18, 19]. Though the facilitators and barriers to health and healthcare access among populations are largely influenced by environmental conditions and occupational health practices, concepts of the Health Belief Model were applied as the focus of the current study to examine the perceptions, attitudes and practices of mineworkers within current structural and systemic drivers. This approach allowed for identification of factors operating at the level of individual workers as well as the system level. A team of 5 researchers (4 US-based, 1 Zambia-based) initially reviewed 4 transcripts and identified recurring elements of participants’ perceptions and narratives about TB and accessing TB services. The research team used these to create codes, such as distrust, severity of TB, and adherence to medication. Codes were organized into a qualitative codebook anchored by components of the Health Belief Model, with deductive codes used to organize inductive codes, and included: perceived threat, cues to action, self-efficacy, perceived barriers/opportunities, and outcomes. The research team coded FGDs using NVivo 11.0 (QSR International) and met weekly to code transcripts to agreement for analysis. These meetings also served as an opportunity to discuss emerging themes and modify the codebook as needed. Following codebook finalization, descriptive codes were applied to the rest of the transcripts to understand the context in which mineworkers and ex-mineworkers were providing feedback.

#### Ethical considerations

This study was reviewed and approved by the Tropical Diseases Research Centre Ethics Review Committee, the Zambian National Health Research Authority, and the Centers for Disease Control and Prevention (USA). All participants provided written, informed consent prior to participation.

## Results

### Study population

**Fig. 1:**
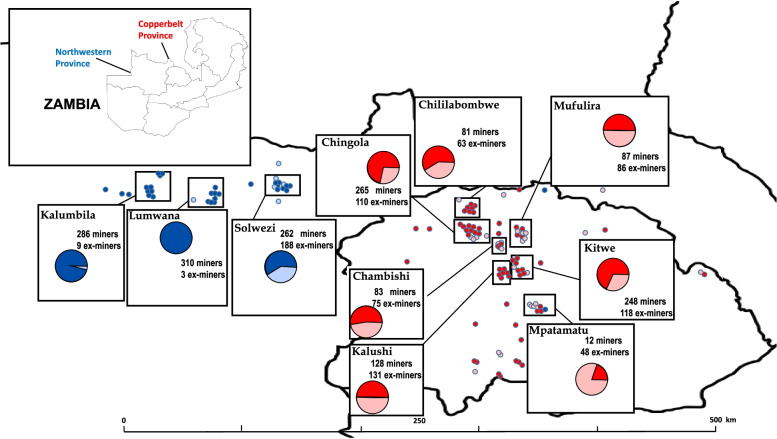
Geographic distribution of participants completing the mineworker KAP survey, by province and employment status

**Fig. 2:**
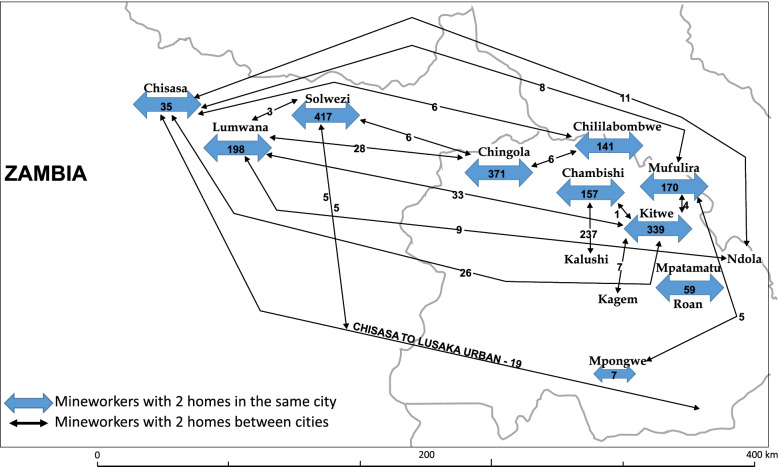
Illustration of number and location of residences among mineworkers in the Copperbelt and North-Western Province who completed the KAP survey and reported ≥ 1 home

Overall, 2,792 participants completed the mineworker KAP survey: 1,711 (61.3%) from the Copperbelt Province and 1,081 (38.7%) from the North-Western Province (Table 1; Fig. 1). The number of participants exceeded the target population due to the manner in which the survey opportunity was advertised; persons presented to complete the survey in large groups and the study team did not want to deny participation and the opportunity to share perspectives. The majority of participants (*n* = 1,956; 70%) were current mineworkers. Ex-mineworkers (*n* = 836) were significantly older than current mineworkers (median age 53 years [Interquartile Range (IQR) 37, 62] vs. 35 years [IQR 31, 42]; *p* < 0.001). Most participants were male (99.0%) and over a quarter (25.3%) held a college degree. Current mineworkers had worked in the mines a median of 9 years (IQR 5, 13); ex-mineworkers had worked for a median of 19 years (IQR 7, 29) (*p* < 0.001). Overall, just over a third (34.4%) of mineworkers and ex-mineworkers were employed by contract vs. directly by the mining company. The proportion of current mineworkers employed by contract was significantly higher than ex-mineworkers (37.9 vs. 26.1%, *p* < 0.001). Copper was the main commodity (94.6%), with an increasing number of persons working in mines over the past decade that produced emeralds or other precious stones (4.9% current mineworkers vs. 0.2% ex-mineworkers). Almost half (48.5%) of ex-mineworkers reported working underground in the mines; however, there was a near-equal distribution across the mining environments of current mineworkers, with approximately one-third each working in underground, surface, or open pit environments. Over one-third (*n* = 681; 34.8%) of current mineworkers reported having more than one residence, most (*n* = 522; 76.6%) among those living in the North-Western Province. Most second homes among mineworkers working in the North-Western Province were located in the Copperbelt Province (69.6%) (Fig. 2).

**Table 1:** Sociodemographic and work characteristics of mineworkers participating in KAP surveys, Zambia (*N* = 2,792). Numbers are n and % unless otherwise specified

	**Total** **(*N* = 2792)**	**Current Mineworker** **(*n* = 1956)**	**Ex-mineworker** **(*n* = 836)**	***p***
Province of Work				
Copperbelt	1711 (61.3)	1077 (55.1)	634 (75.8)	
North-Western	1081 (38.7)	879 (44.9)	202 (24.2)	< 0.001
Age, years – median(IQR)	38 (21, 48)	53 (31, 42)	53 (37, 62)	< 0.001
Gender				
Male	2763 (99.0)	1933 (98.8)	830 (99.3)	
Female	29 (1.0)	23 (1.2)	6 (0.7)	0.27
Highest Education				
None	35 (1.3)	16 (0.8)	19 (2.3)	
Primary	154 (5.5)	61 (3.1)	93 (11.1)	
Junior Secondary	532 (19.1)	312 (16.0)	220 (26.3)	
Senior Secondary	1365 (48.9)	994 (50.9)	369 (44.1)	
College/University	706 (25.3)	571 (29.2)	135 (16.2)	< 0.001
Marital Status				
Never married	381 (13.7)	332 (17.0)	49 (5.9)	
Married/Partner	2308 (82.6)	1573 (80.4)	735 (87.9)	
Divorced, Separated, Widowed	103 (3.7)	51 (2.6)	52 (6.2)	< 0.001
Number of years worked in the mines, median (IQR)	10 (5, 18)	9 (5,13)	19 (7, 29)	< 0.001
Type of employment				
Mining company	1833 (65.7)	1215 (62.1)	618 (73.9)	
Contract	959 (34.4)	741 (37.9)	218 (26.1)	< 0.001
Type of minerals/commodity–most recent mining work				
Copper	2640 (94.6)	1818 (92.9)	822 (98.3)	
Emeralds/precious stones/gems	97 (3.5)	95 (4.9)	2 (0.2)	
Gold or Cobalt	18 (0.6)	13 (0.6)	5 (0.6)	
Not specified/general	37 (1.3)	30 (1.5)	7 (0.9)	< 0.001
Occupational environment – most recent mining work				
Underground	1026 (37.2)	626 (32.4)	400 (48.5)	
Surface (not open pit)	880 (31.9)	604 (31.3)	276 (33.4)	
Open pit	850 (30.9)	701 (36.3)	149 (18.1)	< 0.001
Type of residence^a^				
Family residence	1275 (65.2)	1275 (65.2)	–	–
Work/mining residence only	681 (34.8)	681 (34.8)	–	–

*IQR* interquartile range, *p*-values based on χ^2^ for categorical and Wilcoxon rank-sum test for test of medians

^a^Type of residence utilizing while working in the mines only asked of current mineworkers

Overall, 94 healthcare workers (HCWs) completed the survey (100% of target population): 63 (67%) were from health facilities in the Copperbelt Province and 33% (*n* = 31) were from health facilities in the North-Western Province. The majority (60.6%) of HCWs surveyed worked in government/public health facilities; others worked in private facilities in mining areas (13.8%) or mining-owned facilities (11.7%) (Table 2).

**Table 2:** Occupational characteristics of HCWs who completed KAP surveys, Zambia (*n* = 94)

Characteristic	n (%)
Type of Health Facility	
Mining owned or contracted	11 (11.7)
Government health centre or hospital	57 (60.6)
Private (incl. NGOs)	13 (13.8)
Other	3 (3.2)
Job title	
Clinician or Medical Officer	19 (20.4)
Nurse	18 (19.4)
Enrolled Nurse	20 (21.5)
TB/HIV Coordinator	3 (3.2)
Other	9 (9.6)
Years in Current Position	
< 2 years	19 (20.2)
2–5 years	29 (41.5)
> 5 years	36 (38.3)

Thirty FGDs were conducted with a total of 135 mineworkers and 71 ex-mineworkers during the evaluation period (Table 3). Of these, 22 FGDs were in the Copperbelt Province and eight were in the North-Western Province. Of the 30 FGDs, 18 were with current mineworkers, eight with ex-mineworkers, and four with a combination of current and ex-mineworkers. Four FGDs were purposively conducted with female mineworkers, though they likely represent a small proportion of the total mineworkers in Zambia.

**Table 3:** Sociodemographic characteristics of mineworkers and ex-mineworkers participating in FGDs, Zambia (*N* = 271). Numbers are n and % unless otherwise specified

Focus Group Discussion	Total N	Mineworkers n (%)	Ex-mineworkers n (%)	Males n (%)	Females n (%)	Age Median (IQR)	Years in Mine Median (IQR)
**Copperbelt Province**	**206**	**135 (65.5)**	**71 (34.5)**	**199 (96.6)**	**7 (3.4)**	**39 (29, 48)**	**14 (10, 22)**
Chambishi 1	8	1 (12.5)	7 (87.5)	8 (100)	0	54 (38, 62)	14 (10, 22)
Chambishi 2	10	10 (100)	0	10 (100)	0	36 (28, 50)	6 (3, 17)
Chambishi 3	9	9 (100)	0	9 (100)	0	32 (25, 38)	6 (3, 8.5)
Chililabombwe 1	10	0	10 (100)	10 (100)	0	46 (39, 51)	19 (7, 25)
Chililabombwe 2	8	8 (100)	0	8 (100)	0	39 (35, 44)	15 (10, 18)
Chingola 1	10	0	10 (100)	10 (100)	0	62 (48, 64)	29 (12, 32)
Chingola 2	8	8 (100)	0	8 (100)	0	41 (35, 50)	18 (10, 21)
Chingola 3	10	10 (100)	0	10 (100)	0	29 (27, 31)	6 (5, 8)
Kagem 1	10	10 (100)	0	10 (100)	0	43 (41, 44)	13 (9, 18)
Kagem 2	10	10 (100)	0	10 (100)	0	26 (24, 29)	4 (1, 5)
Kagem 3	10	10 (100)	0	10 (100)	0	30 (26, 39)	4 (2, 6)
Kalulushi 1	8	6 (75.0)	2 (25.0)	8 (100)	0	39 (36, 41)	12 (9, 14)
Kalulushi 2	9	8 (89.0)	1 (11.0)	9 (100)	0	32 (28, 43)	9 (6, 15)
Kitwe 1	10	0	10 (100)	10 (100)	0	56 (53, 61)	21 (20, 26)
Kitwe 2	10	10 (100)	0	10 (100)	0	41 (36, 50)	14 (10, 30)
Kitwe 3	10	10 (100)	0	10 (100)	0	28 (26, 29)	5 (3, 6)
Luanshya 1	11	0	11 (100)	11 (100)	0	43 (39, 49)	15 (14, 28)
Luanshya 2	7	7 (100)	0	7 (100)	0	34 (30, 38)	10 (5, 26)
Mufulira 1	10	0	10 (100)	10 (100)	0	54 (37, 56)	25 (13, 30)
Mufulira 2	11	11 (100)	0	11 (100)	0	42 (40, 47)	11 (8, 20)
Mufulira 3	10	7 (70.0)	3 (30.0)	10 (100)	0	26 (24, 29)	2 (1, 7)
Mufulira 4	7	0	7 (100)	0	7 (100)	30 (26, 31)	3 (2, 5)
**North-Western Province**	**65**	**44 (67.7)**	**21 (32.3)**	**49 (75.3)**	**16 (24.6)**	**33 (30, 37)**	**7 (5, 10)**
Kalumbila 1	10	10 (100)	0	10 (100)	0	34 (38, 42)	3 (1, 7)
Kalumbila 2	7	7 (100)	0	0	7 (100)	29 (24, 32)	6 (4, 11)
Lumwana 1	7	7 (100)	0	7 (100)	0	32 (29, 33)	8 (6, 10)
Lumwana 2	6	6 (100)	0	0	6 (100)	34 (39, 36)	7 (4, 8)
Lumwana 3	11	0	11 (100)	11 (100)	0	34 (31, 38)	3 (2, 8)
Solwezi 1	10	0	10 (100)	10 (100)	0	41 (35, 45)	9 (5, 13)
Solwezi 2	8	8 (100)	0	8 (100)	0	33 (32, 36)	11 (7, 12)
Solwezi 3	6	6 (100)	0	3 (50.0)	3 (50.0)	36 (32, 45)	10 (5, 13)
**TOTAL**	**271**	**179 (66.1)**	**92 (33.9)**	**248 (91.5)**	**23 (8.5)**	**37 (29, 45)**	**9 (5, 15)**

*IQR* interquartile range; *p*-values based on χ^2^ for categorical and Wilcoxon rank-sum test for test of medians

A total of 179 (66.1%) current mineworkers and 92 (33.9%) ex-mineworkers participated in the FGDs. Of these, 248 (91.5%) were male and 23 (8.5%) were female. Eighty-six participants (31.7%) were contract workers while the rest worked directly for the mine. The median age of participants was 37 years (interquartile range [IQR] 29, 45). The median number of years worked in the mining industry was 9 years (IQR 5, 15). The median number of years that the mineworkers and ex-mineworkers spent in school was 12 years. Of the participants, 206 (76%) were married, 64 identified as single (23.6%), and one individual (0.4%) was divorced.

### Mineworkers’ knowledge of TB

Most (94%) mineworker KAP survey participants were able to identify cough as a symptom of TB disease, but few (13.5%) were able to mention more than one symptom of the four that classically characterize TB disease (cough, fever, night sweats, weight loss). The mode of person-to-person respiratory transmission was explained by the majority (81.7%) of participants when asked how TB is spread, and almost all noted that TB could be cured (95%) through drug treatment provided by healthcare workers (99.2%).

### Mineworkers’ self-reported history of TB disease, HIV testing, and current TB symptoms

**Fig. 3:**
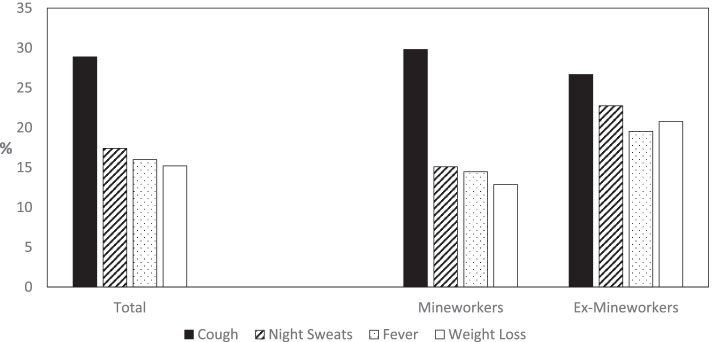
TB Symptoms among Mineworkers and Ex-Mineworkers, Zambia (*n* = 2,792)

Only 5.8% (*n* = 161) of the mineworker KAP survey respondents reported having a previous diagnosis of TB disease; this was significantly higher among ex-mineworkers (12.2%; *n* = 102) than among current mineworkers (3.0%; *n* = 59) (*p* < 0.001). The majority of participants (89.8%; *n* = 2507) had been previously tested for HIV: prior testing was significantly more common among current mineworkers than ex-mineworkers (93.0 vs. 82.3%, *p* =  < 0.001). Almost 30% of respondents had a present cough or cough within 2 weeks prior to the time of the survey (Fig. 3). Other TB symptoms were not infrequent: 12% overall reported two TB symptoms and 7% had three or four symptoms suggestive of TB. Night sweats, fever, and weight loss were significantly more common among ex-mineworkers than current mineworkers (all *p* < 0.001). All mineworkers who reported symptoms at the time of the survey were referred to the health clinic for a complete assessment and TB testing.

### Perceptions about TB and working in the mines

The Zambia 1999 Workers’ Compensation Act prohibits persons from ever working in the mines if they have ever been diagnosed with TB [5, 6]. Only 41.0% of mineworker and ex-mineworker KAP survey respondents knew of the formal policy; however, 70.9% of mineworkers knew they would be prohibited from mine work after a TB diagnosis (Table 4). The majority (76.9%) of mineworkers indicated they would not disclose their TB status to their supervisor; in contrast, 73.8% indicated they would discuss their diagnosis with their spouse.

**Table 4:** Mineworkers’ knowledge and attitudes related to ability to work in the mines if you’ve had TB in the Copperbelt and North-Western Provinces, Zambia (*n* = 2,792). Numbers are n and % unless otherwise specified

	**n (%)**
What is the government policy related to working in the mines	
Cannot work in the mines ever	1146 (41.0)
Can work once non-infectious	632 (22.6)
Can work light duty	49 (1.8)
Don’t know	965 (34.6)
If you have TB, you are allowed to work in the mines	
No	1817 (70.9)
Yes/Yes after treatment	746 (29.1)
If you had TB you would tell your supervisor at work	
No	2148 (76.9)
Yes	644 (23.1)
If you had TB you would tell your spouse	
No	733 (26.3)
Yes	2059 (73.8)

The majority (58.5%) of HCWs were not familiar with the government policy prohibiting persons from working in the mine who had previous or current TB. However, when asked in open-ended questions about key challenges, over half of HCWs (*n* = 32/62; 51.6%) mentioned fear of job loss as a critical barrier to providing timely screening and appropriate care for TB among mineworkers. HCWs mentioned that mineworkers are often denied permission by supervisors to attend medical appointments (*n* = 28/62; 45.2%) and fail to provide accurate contact information (*n* = 12/62; 17.7%) which leads to delayed diagnoses and difficulties in treatment compliance. HCWs cited mineworkers’ fear of job loss as a factor driving these behaviors.

A general fear of job loss also emerged as a primary theme in the mineworker FGDs. This fear was contextualized in various ways, including mining companies prioritizing productivity over the health of their workers. Mineworkers described hiding when they were ill, and continuing to work despite feeling sick to avoid absences on their work record.

A mineworker from Kitwe stated:

“Concerning the company, you will have two thoughts: one, they will fire me from my job once they discover that I have TB; they will fire me. Two, we forget that in future they will come and discover that [I] am sick. So, as a result, you will just be forcing yourself to continue work as you nurse our sickness”

Mineworkers further described feeling like they were just a part of the larger machine of the mine. One current mineworker in Solwezi stated:

**“…**production has affected to a point where, the mine is only thinking of how much [they] will make at the end of the day, and not really concerned about the health of that person, so in that way it is affecting the miner to an extent where, [he’s] just regarded as a spanner or tool of some sort, if it bends… they will just instruct… ‘take it to the workshop… if they hit it and straighten it… bring it back so that we continue using it.’…so they don’t look at the person in terms of total well-being.**”**

Mineworkers also discussed a fear of going to the mine-run health clinics to seek care for TB disease due to potential employment termination. That same fear of job loss was not expressed when choosing to seek TB care at a government-run health clinic. Many mineworkers shared that their colleagues choose to hide that they are taking TB medication from the mining authorities to avoid potential dismissal. Furthermore, several mineworkers detailed their colleagues’ experience of refusing to acknowledge they had a health problem until it caused grave effects.

### Mineworkers’ preferences for care

In the mineworker KAP surveys, 29.1% reported using mining-company facilities, almost half (49.9%) reported using public health facilities, 14.5% used private clinics, and 6.5% used pharmacies or traditional healers as their primary healthcare. Two-thirds (66.6%) reported that they would prefer to seek TB treatment from public health facilities. Very few mentioned desiring to seek TB care at private facilities (4.2%) or pharmacies (0.2%). The preference for public health facilities was slightly higher among contracted mineworkers (77.7%) than those employed directly by the mines (60.8%). Almost all (96.2%) mineworkers and ex-mineworkers relayed that they would seek TB diagnosis and treatment if they suspected they had TB or had symptoms related to TB; 76.6% reported that they would seek care in less than a week.

When asked in the FGDs where they preferred to seek medical care for general health needs versus TB/HIV care, many participants relayed that they would prefer to go to government-run or private clinics for TB and HIV care. Specific barriers to care arose from the FGDs: limited choice in where to seek care, a lack of confidentiality, and low confidence and trust in HCWs. When speaking about the lack of options for healthcare, most mineworkers mentioned that they are required to go to the clinic contracted by the mine and that mine companies would only provide sick leave if the doctor’s note was from the contracted clinic. One ex-mineworker from Solwezi stated:

**“**They are not free, immediately the mining company…if they have not referred you...to ‘XX hospital,’ to go elsewhere, you cannot decide on your own, that now will be outside the mines jurisdiction. You are on your own, so now how is it going to be between you and the company? There will be no coordination, so it’s not easy for a miner to choose to go to government hospitals, it’s not easy.”

Mineworkers reported how the lack of choice in healthcare clinics was often tied directly to a perceived lack of confidentiality as a patient. They reported that mining companies access their patient files and that this access allows them to unfairly influence the course of medical treatment.

A current mineworker from Solwezi stated: “Like me, for example at ‘XX hospital,’ I can’t trust them, because they have introduced an online system…if I am given medicine, even the HR officer will know the kind of drugs I have been given, so in that case there is no privacy.”

### Challenges and solutions for providing TB Care

When HCWs were asked to list key barriers to providing adequate and continuous TB care for mineworkers and ex-mineworkers, the following themes emerged: a) mineworkers’ fear of losing employment/will not disclose TB status at work; b) mineworkers are highly mobile and often do not inform health facilities when they will be away; c) mineworkers often provide inaccurate contact information; and d) mineworkers are often restricted by shift work (outside clinic hours) and/or are denied permissions from work to get care or make appointments. HCWs also offered some key solutions, which included: a) changing the government policy and allowing mineworkers to resume work once non-infectious; b) include mines in the provision of and support for TB care, which would include free access and directly observed therapy (DOT) at work; c) establish health facilities in all mine shafts; d) train all mine health personnel on signs and symptoms of TB and TB care; and e) include regular (quarterly, 6 month, or annual) TB screening for all mineworkers.

## Discussion

This large study of nearly 3000 current and ex-mineworkers and almost 100 healthcare workers identified several key barriers to mineworkers’ receiving timely diagnosis and treatment for TB: mistrust in the mining company, fear of job loss, national policy of not being able to work with current or previous TB, perceived lack of choice in healthcare, and lack of privacy, confidentiality, and trust in healthcare. We revealed major concerns about the conditions and treatment of mineworkers and ex-mineworkers related to health. Mineworkers and ex-mineworkers are quite knowledgeable about TB symptoms and recognize it is curable; however, almost 30% of mineworkers and ex-mineworkers had a cough, and approximately one in five had 2–4 symptoms suggestive of TB at the time of the survey. This observation raises concerns that these individuals may be sick and need care and underscores the important distinction between knowledge and behavior. While the majority of mineworkers reported that they would seek care for TB if they felt as though they may have the illness, findings from the FGDs and HCW KAP surveys suggest that they do not seek care due to distrust in the mining companies and a concern of job loss. Delays in care-seeking behavior and distrust in the healthcare system identified among participants in our study are similar to key findings in a small study conducted among 30 mineworkers and ex-mineworkers in South Africa [20]. Of interest is that these perceptions persist despite very different levels of understanding about clinical symptoms, origin, and transmission of TB disease between the two studies: few of the South African mineworkers or family members understood person-to-person respiratory spread of TB, and most believed it was caused by environmental pollutants or waste near the mines. In this study, both mineworkers and healthcare workers noted that persons are not allowed to work in the mines if they’ve previously had TB and only 23% of mineworkers would tell their supervisors if they suspected they had TB disease. The type of healthcare available was different according to the mechanism of hire (direct hire or contractor), and mineworkers expressed a key desire to have a choice in where they go for healthcare for TB and HIV.

The majority of mineworkers reported they had previously undergone HIV testing, suggesting they are not avoiding or unable to access healthcare, and that they may be receptive to routine HIV counseling and testing and TB screening. A collaborative effort between mines and government might optimize TB and HIV care for mineworkers and enhance TB and HIV control and care.

Our study revealed that over one third of current mineworkers have more than one residence, illustrating potential challenges for TB disease control and continuity of care. The oscillatory migration patterns of mineworkers have been previously recognized as a major contributing factor to ongoing TB disease transmission [2]. Public health and social services to support continuity of care and keep workers and their families safe from additional transmission of disease that was seeded from the workplace are critical to avoiding additional transmission, morbidity and mortality.

Our study had  several strengths and limitations worth noting. We conducted the first survey of this kind among mineworkers in Zambia, which included over 2700 participants across geographic regions in both Copperbelt and North-Western Provinces. This is one of few studies available that includes the perspectives and voices of mineworkers. We derived our target sample size based on 60% current mineworkers and 40% ex-mineworkers, and we did not obtain our target sample of ex-mineworkers. We utilized convenience sampling to recruit for study participation which may limit the generalizability of these findings to the greater population of mineworkers and ex-mineworkers in Zambia. Specifically, the findings may not be representative of all mineworkers or healthcare workers, especially those working in smaller or artisanal mines: some mining companies were not interested in supporting participation or allowing workers to participate in the study. The study used trained research staff to conduct standardized questionnaires and facilitate focus group discussions. Standardized facilitation guides were used and all FGDs were conducted in the local dialect, recorded, and transcribed. Utilizing both quantitative and qualitative methods allowed our findings from the KAPs to be contextualized. Finally, we were not able to collect information or screen for TB infection, disease, or HIV as part of this study. While we recognize this may have been a missed opportunity, due to the need to solicit participation and gain trust among respondents, we focused on establishing relationships with the mining community with hopes that we can improve screening and care access for TB and HIV in the future.

## Conclusion

Mineworkers are a critical population in TB transmission as they are at increased risk for TB due to occupational circumstances, and often undergo transitions in employment and residences. While many mineworkers reported confidence in their ability to access TB services, distrust in the mine company healthcare system emerged as a central theme in the focus group discussions. Activities that demonstrate a commitment from the government and mining companies to improve working conditions and health of employees, build trust in mine company healthcare and offer alternative healthcare choices, promote community TB awareness, and strengthen social support networks among mineworkers may reduce barriers to TB services.

The 1999 Workers’ Compensation Act in Zambia [14] likely results in mineworkers withholding their TB status from their employer for fear of losing employment. This is a key barrier to TB care among mineworkers and has set a tone for distrust and unwillingness of mineworkers to disclose health events to mining administrators, and there is need for immediate repeal of this archaic law and policy. There have been decades of evidence of the oppressive conditions of mineworkers and failure of adequate inspections and oversight to ensure occupational health standards; investment in the health and social rights of mineworking communities – during and following employment – remains an urgent need. Following extensive dissemination of these results among multiple government sectors, mining companies and mineworker unions, and advocacy groups, the Act is being repealed. However, revision of the law is only the first step in facilitating early TB diagnosis and treatment. The advent of the COVID-19 pandemic has presented further scrutiny into occupational health and the susceptibility of persons working in the mines to respiratory illnesses, and has placed responsibilities on mining companies for the health of their workers. Additional work is needed to investigate and mobilize actions to improve working and living conditions of the mining workforce and communities. Advocacy for social change and the rights of mineworkers to work in safe environments and have access to healthcare must be elevated as a priority for the Zambia as part of ensuring mining is sustained as a critical part of the national economy. Controlling TB transmission among mineworkers, ex-mineworkers and their communities will require a commitment by mining companies, occupational health, and the private and public health sectors to ensure patient privacy and facilitate choice of affordable, timely access to high-quality healthcare services, including education, preventive measures, proper protective equipment, and provision of TB care without fear of job loss.

### Disclaimer

The findings and conclusions in this report are those of the authors and do not necessarily represent the official position of PEPFAR, State Department, CDC, USAID, or the U.S. government.

## Data Availability

The datasets used and/or analyzed during the current study are available from the corresponding author on reasonable request.

## Abbreviations

CDC: United States Centers for Disease Control and Prevention
DOT: Directly observed therapy
FGD: Focus Group Discussion
HCW: Healthcare worker
HIV: Human immunodeficiency virus
IQR: Interquartile Range
KAP: Knowledge, attitudes, and practices
PEPFAR: President’s Emergency Plan For AIDS Relief
TB: Tuberculosis
USAID: United States Agency for International Development
